# Effects of Y_2_O_3_/ZrO_2_ Particles on Dielectric Properties and Voltage Resistance of Polyimide Films

**DOI:** 10.3390/ma19122447

**Published:** 2026-06-08

**Authors:** Duoduo Qian, Minjiang Liu, Yuxin Xia, Yan Li, Junjie Yuan, Xiaoyan Xu

**Affiliations:** 1 School of Materials Science and Engineering, Tongji University, Shanghai 201804, China; ddqian@tongji.edu.cn (D.Q.); yuxinxia2022@163.com (Y.X.); yanl@tongji.edu.cn (Y.L.); 2 Shandong Please Drinking Water Co., Ltd., Jinan 250014, China; liuminjiang@vip.sina.com

**Keywords:** polyimide, dielectric film, yttria-stabilized zirconia particle, dielectric properties, breakdown strength

## Abstract

With the advancement of energy storage technology, there have been significantly increased demands for the storage performance and operating temperature of capacitor dielectric materials. As a high-temperature resistant polymer, polyimide (PI) shows great potential for application as a dielectric material. In this study, binary PI composite films with various contents of yttria-stabilized zirconia particles (YZPs) were prepared via in situ polymerization. The results demonstrated that the incorporation of YZPs enhanced the breakdown resistance compared to pure PI films. Specifically, at a YZP content of 8 wt%, the breakdown strength (BDS) of the composite films reached 566 kV·mm^−1^. Although the mechanical strength exhibited a slight reduction, the dielectric properties remained stable, leading to an overall improvement in energy storage performance. Overall, 2 wt% YZPs/PI (with 5 mol% Y_2_O_3_, ZPb2) film is optimal in terms of its mechanical and dielectric properties. This research establishes a solid foundation for the engineering development and industrial implementation of high-performance PI-based polymer dielectric materials.

## 1. Introduction

With the development of environmentally friendly energy exploration and highly efficient energy utilization, it is imperative to develop advanced energy storage technologies [1,2,3,4]. Dielectric capacitors are one of the most important energy storage components [2,3]. Since they possess excellent performance derived from the intermediate dielectric layer, including an ultra-high power density, a low dielectric loss, an all-solid-state structure with a dielectric layer, and a high operating voltage. They are widely used, with practical applications in powertrains, pulse power systems, hybrid electric vehicles and troop weapon systems [1,2,3,4]. However, the common shortcoming lies in the low energy storage density of the dielectric layer [5,6]. Thus, they may not meet the demand in manufacturing large-capacity capacitors. The dielectric layer can be made of various insulating materials, such as ceramic and polymer. Although the traditional ceramic dielectric materials have high dielectric constants, they are brittle, difficult to process and have a low breakdown strength (BDS). In contrast, polymer dielectric materials have significant advantages, including easy processing, good flexibility, light weight, good compatibility and high intrinsic breakdown strength (BDS > 500 kV/mm) [4,5,6]. However, there are two main drawbacks, a low dielectric constant and a restricted operating temperature, in polymer dielectric materials. For example, biaxially oriented polypropylene (BOPP) film is used as a commercial organic film capacitor material with a breakdown field strength of up to 700 kV/mm and an energy storage density of 2.1 J·cm^−3^ [5]. Nevertheless, the heat resistance of aliphatic polymers is relatively poor, the operating temperature of BOPP is limited to about 70 °C and the energy storage density decreases significantly with further increases in temperature [3]. The same restriction also exists in various aromatic polymers with excellent thermal stability. When the temperature exceeds 150 °C, there will be a sharp drop in the energy storage performance [5]. The fundamental reason lies in the fact that high temperatures and strong electric fields have significantly increased the conductive losses of the dielectric materials. Therefore, it is very urgent to overcome the above constraints to adapt to the rapid development of new technological fields.

In this regard, a large number of researchers have focused on composites of inorganic nanofillers and polymer matrix, such as barium titanate (BaTiO_3_) [7,8] with ultra-high dielectric constant (D_k_). Although the introduction of these high dielectric constant fillers can effectively enhance the dielectric constant of the composites, the huge difference between the fillers and the matrix in D_k_ could result in a serious local electric field distortion at the interface of the two phases, leading to a reduction in the BDS and thus impediments to further enhancement of the energy storage density. Various insulating nanomaterials were selected as fillers to be composited with polymer matrix, including titanium series fillers [9] such as barium titanate (BaTiO_3_) and titanium dioxide (TiO_2_) and metal oxide fillers such as zirconia (ZrO_2_) [10]), magnesium oxide (MgO) [11], alumina (Al_2_O_3_) [12,13] and silicon carbide (SiC) [14,15]. It has been found that when the D_k_ of the nanofillers is similar to that of the matrix, the dielectric properties and the energy storage properties could be improved significantly with only a small amount of the nanofillers. Following this trend, core-shell structured nanofillers can establish a D_k_ gradient and simultaneously enlarge the dielectric constant and the BDS at 150 °C [16]. In addition, the researchers also focused on the different dimensions of the fillers [17]. One-dimensional fillers are often prepared by electrospinning with the weaknesses of high energy consumption and low efficiency [18]. Yan et al. [10] prepared one-dimensional fillers of zirconia by the microwave method, but the fillers had a limited effect on the performance due to its small length-to-diameter ratio. Anisotropic two-dimensional materials show great potential for modulation of the properties of the composites such as increasing breakdown field strength, reducing dielectric losses, and improving thermal stability [19]. It was shown that the addition of Barium nitride nanosheets (BNNSs) significantly reduced leakage conductive current, leading to excellent energy storage performance of the composites at 150 °C. However, the synthesis of BNNSs by the liquid-phase stripping method is inefficient. A converse strategy is to utilize fillers with high electron affinity [20]. These materials capture migrating electrons and, through the resulting Coulomb-blocking effect, scatter further electron flow, thus inhibiting migration. The precise control of the filler content is critical to harness this mechanism effectively.

In addition to inorganic fillers, there are also studies on all-organic composite materials, and the composition methods have been expanded from solution blending and in situ polymerization to the construction of multilayer structures [21,22,23,24,25]. Wang et al. [24] prepared the composite films of polyetherimide (PEI) and poly(vinylidene fluoride–trifluoroethylene–chlorotrifluoroethylene) (PVTC) with the layer-by-layer casting method to compare different multilayer structures, including sandwich structure, anti-sandwich structure and directly blending composite. A variety of high-temperature polymers have the potentiality of high-temperature dielectric materials. They have been developed through molecular structure design [26,27,28,29], including the introduction of polar groups such as cyano, sulfone, carbonyl and imino groups, as well as the control of crosslinked network structures. Some of them inherently contain polar groups. For instance, there are imide rings in the molecular structure of polyimide (PI). In recent years, it has shown that PI, with its excellent high-temperature resistance from 100 to 250 °C, is a potential dielectric film for elevated-temperature applications. It is one of the linear dielectric materials with a low dielectric loss, and the dielectric constant will not change significantly with the electric field strength. PI is also characterized by good machinability, good electrical insulation properties, excellent aging resistance and heat resistance, great flexibility and mechanical strength. Ai et al. [9] have demonstrated the positive correlation between the fillers’ bandgap and the BDS of composite films using composites of Al_2_O_3_ (Eg = ~8.6 eV, D_k_ = ~9.5), HfO_2_ (Eg = ~5.8 eV, D_k_ = ~25) and TiO_2_ (~3.0 eV, D_k_ > 30), which provides a reference variable to predict the energy storage performance and the effect of introducing nanofillers. However, it remains to be further studied how this design can be applied universally.

Herein, we present a PI-based composite film. Highly rigid yttria-stabilized zirconia particles (YZPs, Eg = ~5.9 eV, D_k_ = 26–30), designed as fillers, exhibit excellent heat resistance and offer dielectric properties comparable to those of HfO_2_, along with better dispersion. The YZPs/PI composite films were prepared by in situ polymerization and casting with the matrix of polyimide. The effects of the yttria-stabilized zirconia particles (YZPs) on the voltage resistance, dielectric properties, temperature and humidity resistance, and mechanical properties of composite films at low YZP contents were investigated.

## 2. Materials and Methods

### 2.1. Materials

Yttria-stabilized zirconium dioxide (YSZ) particles (YZPs, T3Y and T5Y, average particle size = 0.2–0.3 μm) were obtained from Shandong Sinocera Functional Material Co., Ltd., Dongying, China. T3Y is a kind of YZP with 3 mol% Y_2_O_3_; T5Y is another kind of YZP with 5 mol% Y_2_O_3_. T5Y has lower relative density and hardness than T3Y. Benzene-1,2,4,5-tetracarboxylic dianhydride (PMDA; 98%), 4,4′-oxydianiline (ODA; 98%) and N-methyl-2-pyrrolidinone (NMP) were purchased from Titan Technology Co., Ltd., Shanghai, China. YZPs were dried at 80–100 °C before use. Other chemicals were used as received without further purification.

### 2.2. Preparation of YZPs/PI Films

**Figure 1:**
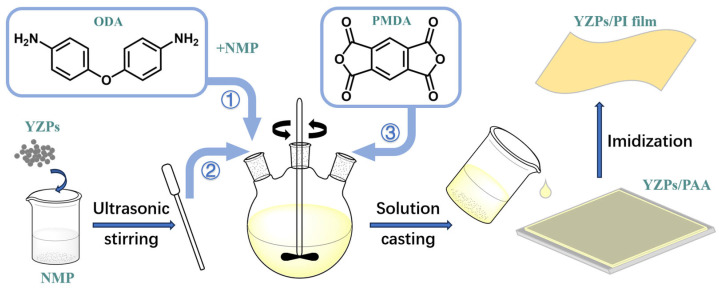
The scheme for the preparation of YZPs/PI film.

**Figure 2:**
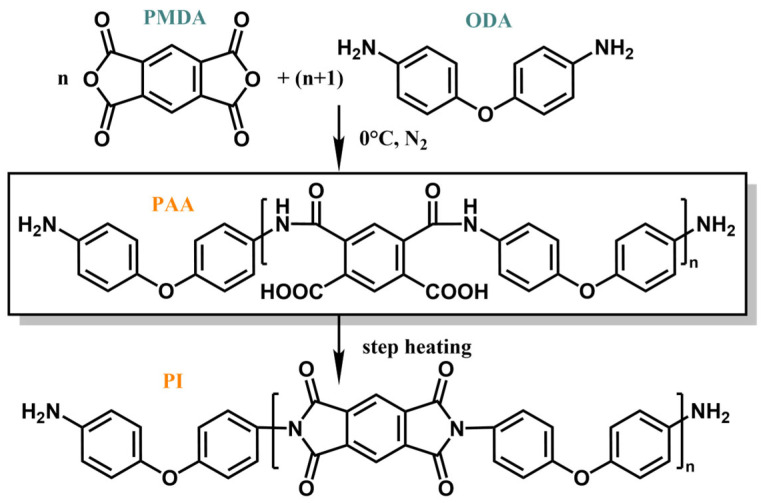
The reaction equation of PMDA and ODA by the thermal imidization method.

The YZPs/PI composite films were prepared by in situ polymerization and casting (Figure 1 and Figure 2) in various weight ratios according to Table 1. Take 2% YZPs-2 for example: ODA (4.025 g, 0.0201 mol) was added into 250 mL three-necked flask with NMP (40–45 g) as a solvent. The dried YZPs (0.168 g) were dispersed in NMP (9 g) by ultrasound and then added into the above mixture after ODA was completely dissolved. Afterwards, PMDA (4.362 g, 0.02 mol) was added bit by bit under 0 °C and nitrogen atmosphere, and the mixture reacted for over 8 h. The mixture has a solid content of 12–15% poly(amic-acid) (PAA). The YZPs/PAA solution was casted on clean and dry glass plate with a blade with uniform and controllable gaps [30]. Volatilization of the solvent and thermal imidization were completed periodically on a heating platform according to the temperature program, which is 50 °C for 4 h, 100 °C for 1 h, 200 °C for 1 h, and 300 °C for 1 h. Finally, the YZPs/PI film was peeled off from the base in deionized water and dried at 80 °C for 12 h. The thickness of the films was controlled in the range of 10–15 μm. The YZPs/PI film samples with a thickness deviation of less than ±2 μm were selected by a micrometer.

### 2.3. Characterization

X-ray diffraction (XRD) experiments were conducted on a D/max 2550 (Rigaku, Tokyo, Japan) diffractometer with Cu Kα radiation (λ = 1.54056 Å) at 40 kV and 40 mA. Fourier-transform infrared spectrogram (FTIR) was recorded by attenuated total reflection (ATR) mode on an INVENIO instrument (Bruker Optics, Karlsruhe, Germany) with a resolution of 0.5 cm^−1^ in the range of 400–4000 cm^−1^. Energy dispersive x-ray spectrogram (EDS) was obtained with a Quanta 200 FEG field emission scanning electron microscope (FEI, Hillsboro, OR, USA) equipped with an energy dispersive spectrometer. The samples were first coated with platinum by sputtering. The tensile test was completed at room temperature using a universal testing machine (CR-10, Transcell, Chicago, IL, USA). The samples were rectangular splines (40 mm × 6 mm). Dielectric properties of the composites were measured using a E5071C vector network analyzer (Agilent, Santa Clara, CA, USA) at 10 GHz at room temperature. DC breakdown strength (BDS) was evaluated using a MS2677A-I breakdown strength tester in silicone oil at room temperature by applying a DC voltage ramp at a rising rate of 200 V·s^−1^ and a limit current of 5 mA. Over 10 samples were measured for each condition. Thermogravimetric analysis (TGA) was performed from room temperature to 800 °C using a SDT Q600 instrument (TA Instruments, New Castle, DE, USA) at a heating rate of 10 °C·min^−1^ in N_2_ flow (20 mL·min^−1^). Water absorption was tested according to ISO 62:2008 [31].

## 3. Results and Discussion

### 3.1. Dispersed Microstructure of YZPs/PI Films

**Figure 3:**
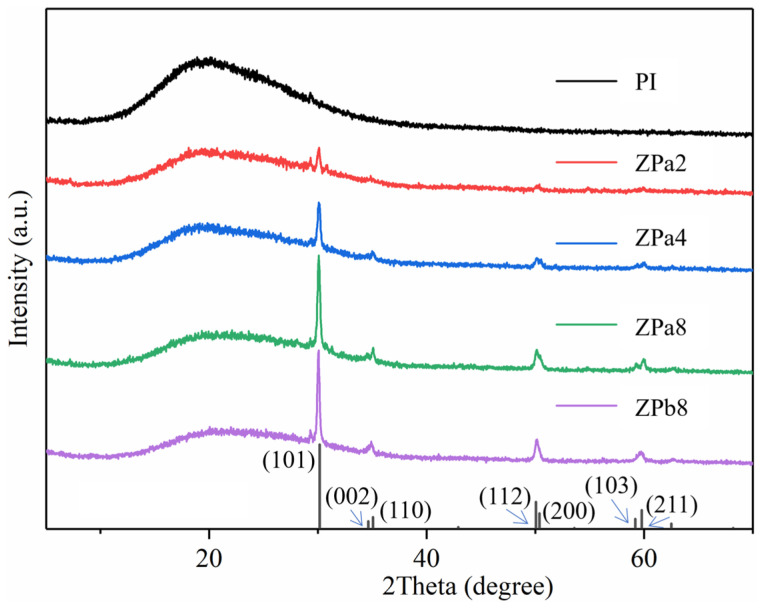
XRD patterns of PI and YZPs/PI composite films.

The XRD patterns of the YZPs/PI films are shown in Figure 3. The diffractogram of the pure PI film presents only a broad region (around 20°), characteristic of a non-crystalline material. Meanwhile, the YZPs/PI films have several sharp peaks with a synchronized 2θ position. According to the XRD pattern of the zirconium dioxide (PDF#89-7710), there are seven peaks from 30° to 60° corresponding to the (101), (002), (110), (112), (200), (103) and (211) plane, respectively. The higher the YZP content, the higher the sharp diffraction peak. When YZPs are added to the PI, the characteristics of the PI matrix do not change and the peaks of the YZPs become more obvious. It is evidenced that the crystal structure of the PI matrix is not affected when the YZPs are integrated into the PI matrix.

**Figure 4:**
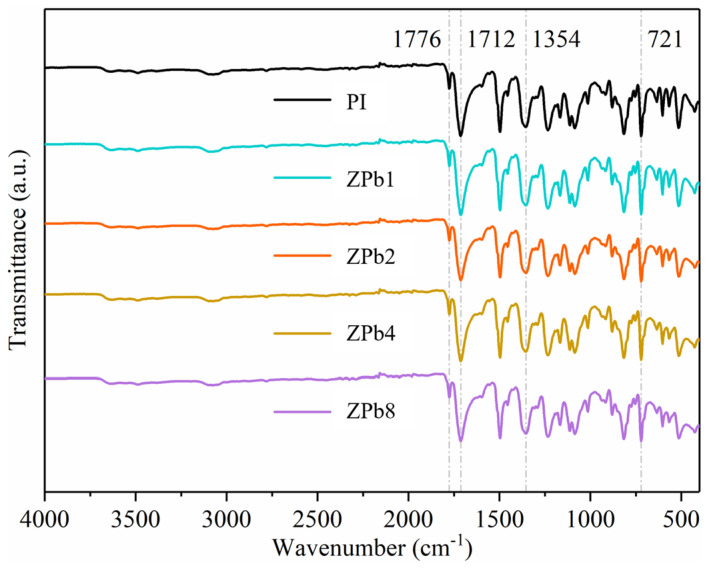
ATR-FTIR spectra of PI and YZPs/PI composite films.

As illustrated in Figure 4, the FTIR spectra of the PI and YZPs/PI composite films are almost identical. The appearance of bands at 1776 cm^−1^ and 1712 cm^−1^ (asymmetric and symmetric C=O stretching), 1354 cm^−1^ (C-N stretching) and 721 cm^−1^ (C=O bending) is related to the imide ring. It suggests that the chemical structure of the PI matrix is not affected by the incorporated YZPs. The intensity of the broad peak at 521 cm^−1^, attributed to asymmetric Zr–O stretching [32], increases with increasing YZP content. The data reveal that the addition of YZPs in the matrix should be a physical dispersion rather than a chemical reaction.

**Figure 5:**
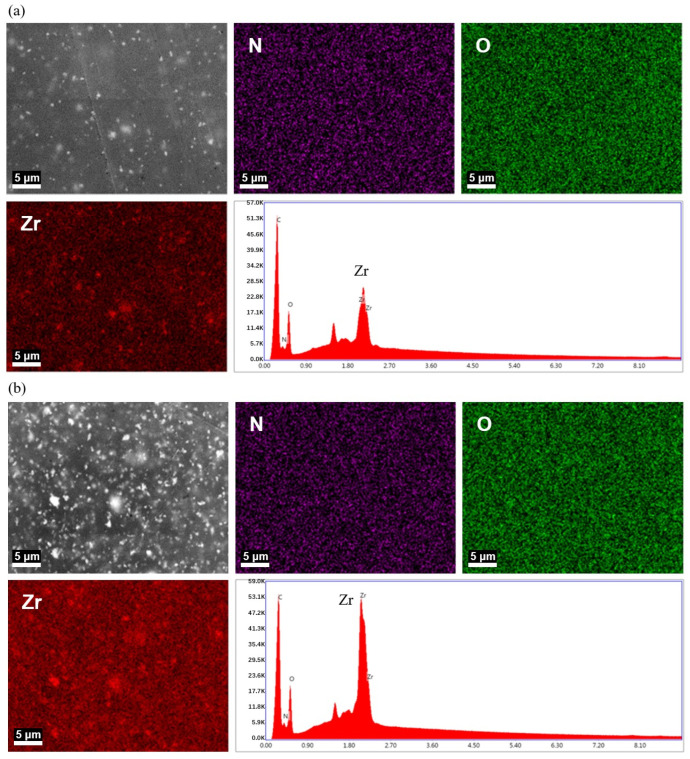
Energy dispersive X-ray spectroscopy image of YZPs/PI composite films. (**a**) ZPb2; (**b**) ZPb8.

The elemental analysis was obtained using an energy dispersive spectrometer, which was configured on a field emission scanning electron microscope. The energy dispersive X-ray spectroscopy images of the YZPs/PI composite films are illustrated in Figure 5. The dark-grey areas in the Figure are the PI matrix and the white parts are the YZPs fillers. The images show a uniform dispersion of the N and O elements, while the element Zr is not evenly distributed. It is obvious that the ZPb8 film has more Zr content than the ZPb2 film. Equally impressively, the element Zr forms more obvious aggregates at high YZP content. The randomly dispersed YZPs are estimated to be almost less than 1 µm in size. The uneven distribution may affect the actual performance.

### 3.2. Mechanics Properties of YZPs/PI Films

**Figure 6:**
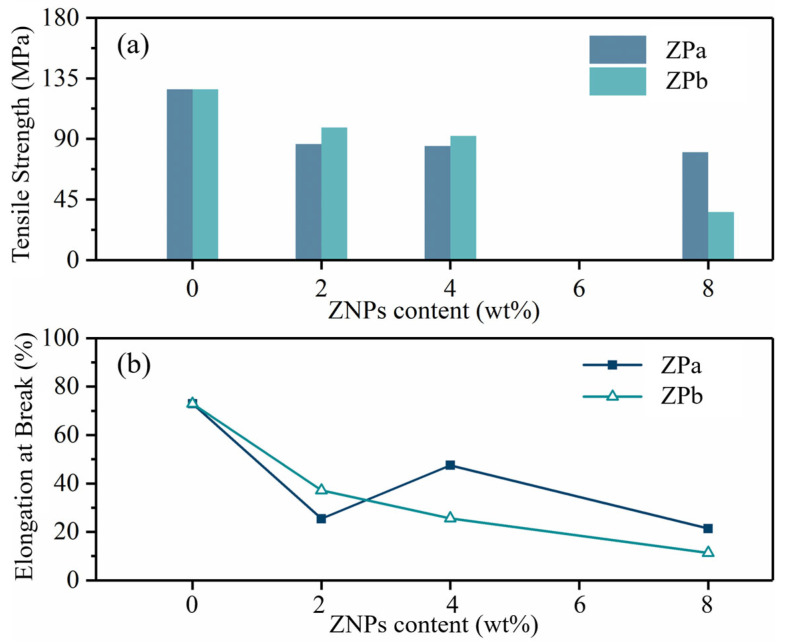
Variations for (**a**) tensile strength and (**b**) elongation at break of YZPs/PI composite films with weight fraction of YZPs.

The mechanical properties of the different YZPs/PI films are summarized in Figure 6. Both the tensile strength (127 MPa) and the elongation at break (73%) of the pure PI films are better than those of the YZPs/PI films. For both the ZPa and ZPb series, the tensile strength decreases gradually with increasing YZP content. YZPs, as a kind of rigid particle with high hardness, can be stress concentration points [33] and damage the uniformity of the polyimide matrix. YZPs may disrupt the interactions of molecular chains in matrix. In the plastic deformation stage, the extension of the PI chain is hindered by the rigid YZPs, causing inner defects and accelerating the fracture, which macroscopically shows a decrease in tensile strength. Secondly, although the in situ polymerization method can help YZP fillers to achieve good dispersion in the PI matrix, there are still compatibility problems between the fillers and the matrix, such as some degree of phase separation phenomenon. Combined with the FTIR result, this shows that the particles are only physically dispersed. The absence of strong interfacial bonding may weaken the filler–matrix interface [34], making it prone to debonding under mechanical stress and leading to the observed reduction in mechanical performance. It is obvious that the more the YZP content, the more significant the compatibility problem. When the YZP content is the same, the tensile strength of the ZPb series is greater than the ZPa series. The ZPb fillers have lower relative density and hardness than the ZPa fillers. The greater the hardness and the relative density of the fillers, the greater the damage to the tensile strength when they act as a stress concentration point.

The trend of elongation at break is similar to that of tensile strength. The compatibility problems and the stress concentration cause the decreased ductility. The YZPs filled the gap in the chains in the matrix, so it is more difficult for the YZP/PI film to deform under tensile force, and then the elongation at break decreases.

### 3.3. Dielectric Properties of YZPs/PI Films

**Figure 7:**
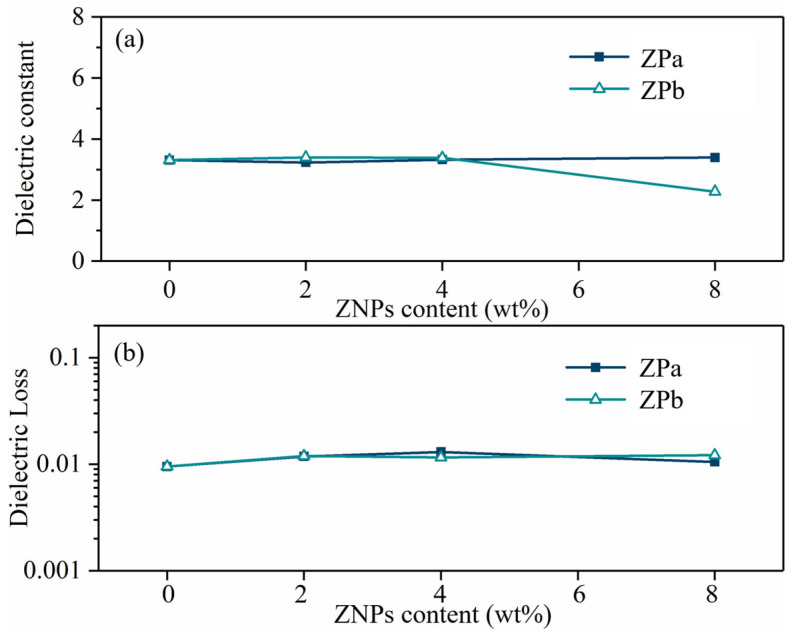
Variations for (**a**) dielectric constant and (**b**) dielectric loss of YZPs/PI composite films (RT, 10 GHz) with weight fraction of YZPs.

Figure 7 shows the variations in the dielectric constant (D_k_) and the dielectric loss (D_f_) of the YZPs/PI films with the YZP content at room temperature and a test frequency of 10 GHz. The detailed data are summarized in Table 2 below. The dielectric constant of the composite film is basically the same as that of the pure PI films (D_k_ = 3.31) at the high frequency. In general, there is an interface between the filler and the organic PI phase, leading to the generation of interfacial polarization and an increase in the dielectric constant, especially for the fillers with a high dielectric loss. However, when YZPs are introduced into the PI matrix, the D_k_ is relatively stable; the main reason may be that the dielectric constant of zirconium dioxide is only an order of magnitude different from that of the PI matrix, and a small amount of YZPs will not cause significant changes in the dielectric constant of the composite films [9,35]. At the same time, the dielectric constant of the YZPs/PI film is not only affected by that of the filler itself and the YZP content but also it is limited by the size and shape of the filler and the interaction between the two phases. In this study, the YZPs have an average particle size of 0.2–0.3 μm, which may hinder the promotion of the D_k_. The dielectric constant of the ZPb8 film (D_k_ = 2.28) is lower than the others. The differences in the dielectric constant and the dielectric loss may partly arise from measurement errors or from variations in the homogeneity of the YZP distribution, especially at higher filler concentrations where agglomeration becomes more likely. The abnormally reduced value of ZPb8 may be related to the bad dispersity of the fillers. At this time, YZPs are more likely to agglomerate. Thus, the uniformity of the film may be destroyed, and defects such as tiny pores will be introduced, resulting in a decrease in the dielectric constant.

In addition, all the dielectric losses of the YZPs/PI films are low, and the change in the dielectric loss is only in the same order of magnitude (about 0.01). This demonstrated that the addition of YZPs with close filler content will not adversely affect the dielectric properties of PI films.

### 3.4. Voltage Resistance of YZPs/PI Films

**Figure 8:**
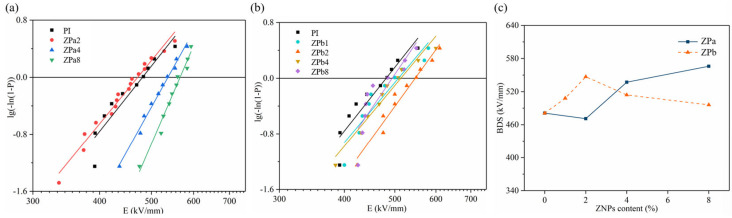
Weibull plot of YZPs/PI composite films. (**a**) ZPa series; (**b**) ZPb series. (**c**) Weibull-fitted breakdown strength of YZPs/PI composite films.

Breakdown strength (BDS) is one of the most important parameters to compare the energy storage performance of composite films. In this paper, the DC breakdown strength of the YZPs/PI films at room temperature with different YZP content (as shown in Figure 8) was analyzed by a two-parameter Weibull statistic distribution function [36] described in Equation (1):

(1) $$P\left(E\right)=1-exp(-{\left(\frac{E}{E_{b}}\right)}^{\beta })$$

where *P(E)* represents the cumulative probability of electric failure, *E* is the measured breakdown field strength, the Weibull breakdown strength *E_b_* is the field strength at the cumulative failure probability of 63.2%, and *β* is the shape parameter to evaluate the dispersivity of the breakdown strength data. At least 10 samples were measured for each Weibull fitting. The breakdown strength and shape parameter *β* of the YZPs/PI films are listed in Table 3. As summarized in Table 3, the shape parameter *β* is greater than eight in all cases. It was proved that the BDS value has a good reflection on the voltage resistance of the YZPs/PI films.

Apparently, in order to improve the voltage resistance of PI films by adding YZPs, a higher YZP content is not better, as shown in Table 3. The maximum BDS of the ZPb series is 547 kV·mm^−1^ when the YZP content is 2 wt%, while that of the ZPa series reaches to 566 kV·mm^−1^ at the YZP content of 8 wt%. For the ZPb series, when the YZP content reaches 8 wt%, the BDS is the lowest, but it is still slightly higher than that of the pure PI film (481 kV·mm^−1^). Compared with previously reported polyimide-based dielectric composites [9,14], the YZPs/PI films in this work achieve a comparable increment in breakdown strength while maintaining good dielectric constant matching. The observed decrease in the BDS may be attributed to poor filler dispersibility. The well-dispersed YZPs may be efficient in acting as barriers against charge injection from the electrodes and the growth of electrical trees in the dielectric film and inhibiting the formation of a conductive path under a high electric field [21]. In addition, the interfaces between the YZPs and the PI matrix can also contribute to the higher BDS of the ZPb series compared to the pure PI film. This is because they could impede charge conduction in the YZPs/PI films as effective electron scatters and trapping centers [24,25]. Concomitantly, YZPs have a close D_k_ to the PI matrix, which may avoid the significant distortion of local fields to some extent, especially with a suitable and low YZP content. The enhanced distortion of local fields is also the main reason for the rapidly reduced BDS at high YZP content such as in ZPb8. Notably, the optimal YZP content for BDS differs between the two filler types. The harder ZPa fillers cause more severe stress concentration, suppressing the BDS at low loadings. Therefore, a higher loading (8 wt%) is required for the beneficial interface effects to compensate for the mechanical disruption. A further increase in the YZP content results in not only poor film forming quality and reduced flexibility but also decreased BDS.

### 3.5. Environmental Performance Properties of YZPs/PI Films

**Figure 9:**
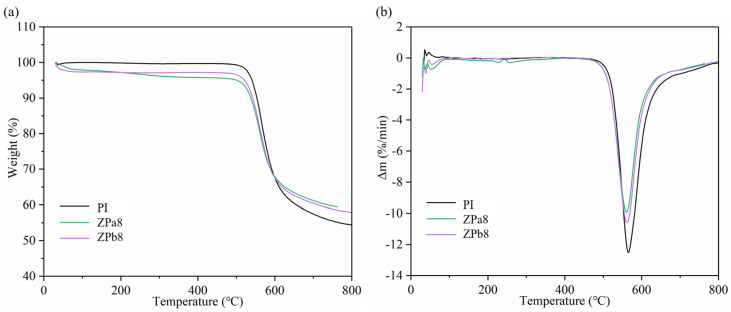
(**a**) TGA curves and (**b**) DTG curves of YZPs/PI composite films.

Some special operating environments should be considered for the dielectric film in practical applications, including high temperature and high humidity. As the matrix of the films, PI has excellent heat resistance. Inorganic fillers (YZPs) have a decomposition temperature of more than 1000 °C. In order to show the effects of the YZPs on the thermal stability of the composite films, ZPa8 and ZPb8, with higher filler contents, were tested by thermogravimetric analysis and compared with the commercial PI films (as shown in Figure 9). There are two weight loss steps in the curves of ZPa8 and ZPb8 in Figure 9a. In the first tiny stage, the weight loss before 100 °C is likely due to the evaporation of water in the composite films. The second step is the main one, and the weight loss between 500 °C and 700 °C is mainly the result of the decomposition of the polyimide. Therefore, all the DTG curves in Figure 9b of the YZPs/PI films have only one clearly visible peak. Both ZPa8 and ZPb8 have similar thermal stabilities when compared with the pure PI film and will not decompose significantly until 500 °C.

The data from the TGA test is listed in Table 4. It is shown that the 5% weight loss temperature of all the films is above 500 °C, and the weight loss between 100 to 750 °C of the pure PI film is about 8% more than those of ZPa8 and ZPb8, which matches the fact of their 8 wt% residual filler addition. Furthermore, the maximum decomposition rate temperatures of ZPa8 and ZPb8 are comparable, and their 10% weight loss temperatures are also relatively close. With the addition of the YZPs, the thermal stability of the composite films will not be reduced. It is demonstrated that the good thermal stability may not be influenced by the different kinds of YZPs with various relative densities and hardnesses, and the in situ polymerization method can be used to promote dispersion without affecting the structure of the matrix.

**Figure 10:**
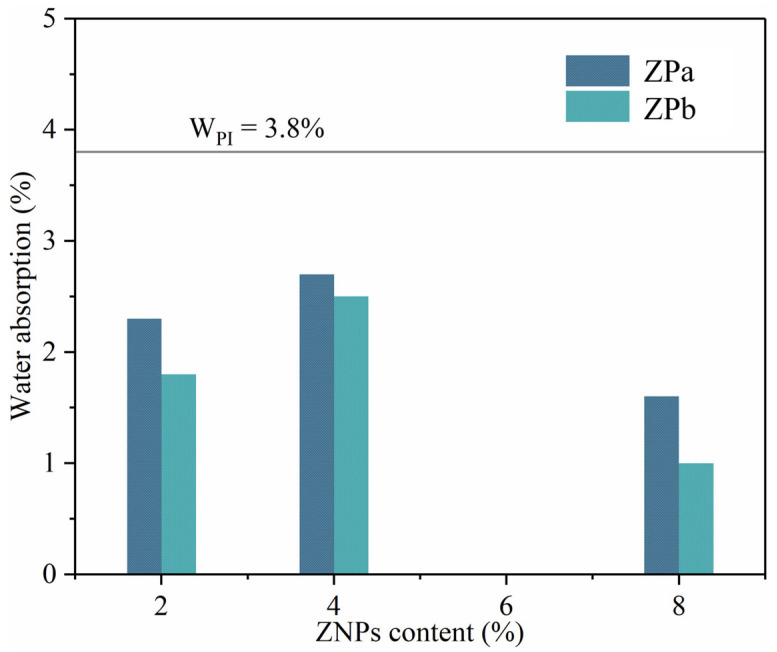
Water absorption of YZPs/PI composite films.

In parallel, it is important for the dielectric materials to reduce the water absorption in humid environments. Figure 10 shows the water absorption of the YZPs/PI films over 24 h. The data reveal that the composite films have lower water absorption than the pure PI film, and actually they all remain at a similarly low level of below 4%. The water absorption of ZPb8 is only 1.0%. On the one hand, as for the PI matrix, there are a large number of polar imide rings in the PI structure. These imide rings lead to the tight attraction and the close stacking of the molecular chains in the matrix. Thus, it is difficult to absorb water into such a compact structure, and therefore it shows low water absorption. On the other hand, the addition of the YZPs further reduces the water absorption due to the interfaces between the YZPs and the PI matrix in the composite film. The dispersed YZPs create a more tortuous pathway for moisture diffusion, extending the diffusion length and effectively slowing water ingress. The YZPs occupy some of the free volume originally present in the PI matrix, thereby inhibiting water penetration. The series of ZPb films have generally lower water absorption than that of the ZPa series. In general, the low water absorption, especially for the composite films with YZPs, is meaningful, because the ionization of inner water will lead to greater conductivity and higher dielectric loss of the films, which will directly affect working life and stability.

In summary, compared with previously reported polyimide-based composites [8,9,10,14], the YZPs/PI films achieve a comparable enhancement in breakdown strength while maintaining a favorable dielectric constant and low loss (Table 5). This balanced performance underscores the advantage of incorporating rigid YZP fillers. Thus, the YZPs/PI system represents a robust platform for dielectrics requiring both thermal resilience and electrical stability.

## 4. Conclusions

In this work, two series of YZPs/PI composite films with various YZP contents were prepared by the in situ polymerization method. The films with YZP fillers show improved voltage resistance. At a YZP content of 8 wt%, the BDS of ZPa8 reaches 566 kV·mm^−1^. The ZPb2 film, which exhibits superior overall performance, also achieves a breakdown voltage of 547 kV·mm^−1^. Compared with the pure PI films, the addition of YZPs improved the breakdown resistance. The dielectric properties were kept stable so that the energy storage performance would improve with the addition of YZPs. While the addition of YZPs degrades flexibility and mechanical properties to different extents, it simultaneously reduces water absorption and preserves high-temperature stability. Therefore, this work lays a solid foundation for the further development and modification of PI polymer dielectric materials for practical applications.

## Figures and Tables

**Table 1:** Components of YZPs/PI films.

Label	YZP Type	YZP Content (wt%)
PI	-	0
ZPa2	T3Y	2
ZPa4	T3Y	4
ZPa8	T3Y	8
ZPb1	T5Y	1
ZPb2	T5Y	2
ZPb4	T5Y	4
ZPb8	T5Y	8

**Table 2:** The dielectric constant and dielectric loss of YZPs/PI films.

Film Type	D_k_	D_f_
PI	3.31	0.0095
ZPa2	3.23	0.0118
ZPa4	3.33	0.0130
ZPa8	3.39	0.0105
ZPb2	3.40	0.0119
ZPb4	3.38	0.0116
ZPb8	2.28	0.0121

**Table 3:** The breakdown strength and Shape parameter *β* of YZPs/PI films.

Films Type	BDS (kV/mm)	*β*
PI	481	9.43
ZPa2	471	9.13
ZPa4	537	13.49
ZPa8	566	17.34
ZPb1	508	8.89
ZPb2	547	10.31
ZPb4	514	8.97
ZPb8	496	11.10

**Table 4:** TGA data of YZPs/PI films.

Item	PI	ZPa8	ZPb8
T_5_ [°C]	541	503	527
T_10_ [°C]	554	540	545
T_max_ [°C]	567	560	563
Residual weight at 750 °C (%)	56.2	59.7	58.8
Weight loss of 100–750 °C (%)	44.5	38.2	38.5

**Table 5:** Comparison of the properties of YZPs/PI films with other reported PI-based hybrid films at room temperature.

Reference	D_k_	D_f_	BDS (kV/mm)
1 vol% BTNFs/PI [8]	3.7 @ 1 kHz	0.025 @ 1 kHz	550
PI [9]	3.33 @ 1 kHz	0.02 @ 1 kHz	314
1 vol% TiO_2_/PI [9]	3.58 @ 1 kHz	0.03 @ 1 kHz	340
5 vol% HfO_2_/PI [9]	3.76 @ 1 kHz	0.03 @ 1 kHz	397
7 vol% Al_2_O_3_/PI [9]	4.02 @ 1 kHz	0.04 @ 1 kHz	422
10 wt% ZrO_2_/PI [10]	5.1 @ 10 Hz	0.05 @ 10 Hz	Not Reported
3 wt% SiC_p_/PI [14]	4.3 @ 10 kHz	0.02 @ 10 kHz	Not Reported
3 wt% SiC_w_/PI [14]	5.6 @ 10 kHz	0.01 @ 10 kHz	Not Reported
PI (This work)	3.31 @ 10 GHz	0.0095 @ 10 GHz	481
ZPb2 (This work)	3.40 @ 10 GHz	0.0119 @ 10 GHz	547
ZPa8 (This work)	3.39 @ 10 GHz	0.0105 @ 10 GHz	566

## Data Availability

The original contributions presented in this study are included in the article. Further inquiries can be directed to the corresponding authors.

## Abbreviations

The following abbreviations are used in this manuscript:

PI	Polyimide
YZPs	Yttria-stabilized zirconia particles
BDS	Breakdown strength
BOPP	Biaxially oriented polypropylene
BNNSs	Barium nitride nanosheets
PEI	Polyetherimide
PVTC	Poly(vinylidene fluoride–trifluoroethylene–chlorotrifluoroethylene)
YSZ	Yttria-stabilized zirconium dioxide
T3Y	A kind of YZPs with 3 mol% Y_2_O_3_
T5Y	A kind of YZPs with 5 mol% Y_2_O_3_
PMDA	Benzene-1,2,4,5-tetracarboxylic dianhydride, pyromellitic dianhydride
ODA	4,4′-Oxydianiline
NMP	N-methyl-2-pyrrolidinone
PAA	Poly(amic-acid)
XRD	X-ray diffraction
FTIR	Fourier-transform infrared spectrogram
ATR	Attenuated total reflection mode
EDS	Energy dispersive X-ray spectrogram
TGA	Thermogravimetric analysis
BTNF	BaTiO_3_ nanofibers (large aspect ratio)

## References

[1] Zhang T., Sun H., Yin C., Jung Y.H., Min S., Zhang Y. (2023). Recent progress in polymer dielectric energy storage: From film fabrication and modification to capacitor performance and application. Prog. Mater. Sci..

[2] He Q., Sun K., Shi Z., Liu Y., Fan R. (2023). Polymer dielectrics for capacitive energy storage: From theories, materials to industrial capacitors. Mater. Today.

[3] Luo H., Wang F., Guo R., Zhang D., He G., Chen S. (2022). Progress on polymer dielectrics for electrostatic capacitors application. Adv. Sci..

[4] Saxena P., Shukla P. (2021). A comprehensive review on fundamental properties and applications of poly(vinylidene fluoride) (PVDF). Adv. Compos. Hybrid Mater..

[5] Zha J.W., Xiao M., Wan B., Wang X., Dang Z.M., Chen G. (2023). Polymer dielectrics for high-temperature energy storage: Constructing carrier traps. Prog. Mater. Sci..

[6] Huai K., Robertson M., Che J., Wang Q., Liu X., Xia Y. (2023). Recent progress in developing polymer nanocomposite membranes with ingenious structures for energy storage capacitors. Mater. Today Commun..

[7] Jiang Y., Zhang X., Shen Z., Li X., Yan J., Li B.W. (2020). Ultrahigh breakdown strength and improved energy density of polymer nanocomposites with gradient distribution of ceramic nanoparticles. Adv. Funct. Mater..

[8] Hu P., Sun W., Fan M., Qian J., Jiang J., Dan Z. (2018). Large energy density at high-temperature and excellent thermal stability in polyimide nanocomposite contained with small loading of BaTiO_3_ nanofibers. Appl. Surf. Sci..

[9] Ai D., Li H., Zhou Y., Ren L., Han Z., Yao B. (2020). Tuning nanofillers in in situ prepared polyimide nanocomposites for high-temperature capacitive energy storage. Adv. Energy Mater..

[10] Yan Y.Z., Park S.S., Moon H.R., Zhang W.J., Yuan S., Shi L. (2021). Thermally robust zirconia nanorod/polyimide hybrid films as a highly flexible dielectric material. ACS Appl. Nano Mater..

[11] Wang P., Guo Y., Zhou D., Li D., Pang L., Liu W. (2022). High-temperature flexible nanocomposites with ultra-high energy storage density by nanostructured MgO fillers. Adv. Funct. Mater..

[12] Chen Y., Zhang D., Wu X., Wang H., Zhang C., Yang W. (2017). Epoxy/α-alumina nanocomposite with high electrical insulation performance. Prog. Nat. Sci. Mater..

[13] Zhang T., Chen X., Thakur Y., Lu B., Zhang Q., Runt J. (2020). A highly scalable dielectric metamaterial with superior capacitor performance over a broad temperature. Sci. Adv..

[14] Zhang Z.X., Yang S.K., Shen J.W., Yang J., Bian J., Zhang A.P. (2022). Enhanced mechanical, thermal and dielectric properties of polyimide nanocomposites containing SiCp (SiCw) nanofillers for high energy-storage applications. J. Polym. Res..

[15] Cao X., Zhao W., Gong X., Zhang D., Su Q., Zha J. (2021). Mussel-inspired polydopamine functionalized silicon carbide whisker for PVDF composites with enhanced dielectric performance. Compos. Part A-Appl. Sci. Manuf..

[16] Ai D., Wu C.L., Han Y.T., Chang Y., Xie Z.L., Yu H. (2025). Polymer nanocomposites with concurrently enhanced dielectric constant and breakdown strength at high temperature enabled by rationally designed core-shell structured nanofillers. J. Mater. Sci. Technol..

[17] Wang G., Huang X., Jiang P. (2015). Tailoring dielectric properties and energy density of ferroelectric polymer nanocomposites by high-k nanowires. ACS Appl. Mater. Inter..

[18] Pan Z., Zhai J., Shen B. (2017). Multilayer hierarchical interfaces with high energy density in polymer nanocomposites composed of BaTiO_3_@TiO_2_@Al_2_O_3_ nanofibers. J. Mater. Chem. A.

[19] Ma G.X., Lei C.S., Liu T., Liu Y.Y., Li W.J., Zhang H. (2022). Improved dielectric and energy storage properties of polymer composites with BNNSs/AgNPs hybrid nanofiller. Mater. Technol..

[20] Wang H., Luo H., Liu Y., Wang F., Peng B., Li X.N. (2024). Improved energy density at high temperatures of FPE dielectrics by extreme low loading of CQDs. Materials.

[21] Jiang J., Shen Z., Qian J., Dan Z., Guo M., Lin Y. (2019). Ultrahigh discharge efficiency in multilayered polymer nanocomposites of high energy density. Energy Stor. Mater..

[22] Liang X., Yu X., Lv L., Zhao T., Luo S., Yu S. (2020). BaTiO_3_ internally decorated hollow porous carbon hybrids as fillers enhancing dielectric and energy storage performance of sandwich-structured polymer composite. Nano Energy.

[23] Guo R., Luo H., Yan M., Zhou X., Zhou K., Zhang D. (2021). Significantly enhanced breakdown strength and energy density in sandwich-structured nanocomposites with low-level BaTiO_3_ nanowires. Nano Energy.

[24] Wang C., He G., Chen S., Zhai D., Luo H., Zhang D. (2021). Enhanced performance of all-organic sandwich structured dielectrics with linear dielectric and ferroelectric polymers. J. Mater. Chem. A.

[25] Zhang T., Sun Q., Kang F., Wang Z., Xue R., Wang J. (2022). Sandwich-structured polymer dielectric composite films for improving breakdown strength and energy density at high temperature. Compos. Sci. Technol..

[26] Zhu T., Yu Q., Zheng W., Bei R., Wang W., Wu M. (2021). Intrinsic high-k -low-loss dielectric polyimides containing ortho-position aromatic nitrile moieties: Reconsideration on Clausius-Mossotti equation. Polym. Chem..

[27] Tang Y.D., Yao H.Y., Xu W.H., Zhu L.X., Zhang Y.H., Jiang Z.H. (2022). Side-chain-type high dielectric-constant dipolar polyimides with temperature resistance. Macromol. Rapid Commun..

[28] Liu X.J., Zheng M.S., Wang G., Zhang Y.Y., Dang Z.M., Chen G. (2022). High energy density of polyimide films employing an imidization reaction kinetics strategy at elevated temperature. J. Mater. Chem. A.

[29] Wu Z.Q., Peng Y.W., Song Y., Liang H.Y., Gong L., Liu Z.G. (2023). Polyimide dielectrics with cross-linked structure for high-temperature film capacitors. Mater. Today Energy.

[30] Zhang B., Chen X.M., Pan Z., Liu P., Mao M.M., Song K.X. (2023). Superior high-temperature energy density in molecular semiconductor/polymer all-organic composites. Adv. Funct. Mater..

[31] (2008). Plastics—Determination of Water Absorption.

[32] Le A., Steimle T.C., Gupta V., Rice C.A., Maier J.P., Lin S.H. (2011). The visible spectrum of zirconium dioxide, ZrO_2_. J. Chem. Phys..

[33] Wang Y.K., Zhu G.M., Tang Y.S., Xie J.Q., Liu T.T., Liu Z. (2014). Mechanical and shape memory behavior of chemically cross-linked SBS/LDPE blends. J. Polym. Res..

[34] Longun J., Walker G., Iroh J.O. (2013). Surface and mechanical properties of graphene–clay/polyimide composites and thin films. Carbon.

[35] Luo S.B., Shen Y.B., Yu S.H., Wan Y.J., Liao W.H., Sun R. (2017). Construction of a 3D-BaTiO_3_ network leading to significantly enhanced dielectric permittivity and energy storage density of polymer composites. Energy Environ. Sci..

[36] Rytoluoto I., Lahti K. (2013). New approach to evaluate area-dependent breakdown characteristics of dielectric polymer films. IEEE Trans. Dielectr. Electr. Insul..

